# Neglect-Like Effects on Drawing Symmetry Induced by Adaptation to a Laterally Asymmetric Visuomotor Delay

**DOI:** 10.3389/fnhum.2018.00335

**Published:** 2018-08-28

**Authors:** Chen Avraham, Guy Avraham, Ferdinando A. Mussa-Ivaldi, Ilana Nisky

**Affiliations:** ^1^Biomedical Engineering, Ben-Gurion University of the Negev, Beersheba, Israel; ^2^Zlotowski Center for Neuroscience, Ben-Gurion University of the Negev, Beersheba, Israel; ^3^Department of Psychology, University of California, Berkeley, Berkeley, CA, United States; ^4^Department of Physiology and Department of Biomedical Engineering, Northwestern University, Evanston, IL, United States

**Keywords:** visuomotor delay, space-variant delay, reaching, drawing, adaptation, transfer, hemispatial neglect

## Abstract

In daily interactions, our sensorimotor system accounts for spatial and temporal discrepancies between the senses. Functional lateralization between hemispheres causes differences in attention and in the control of action across the left and right workspaces. In addition, differences in transmission delays between modalities affect movement control and internal representations. Studies on motor impairments such as hemispatial neglect syndrome suggested a link between lateral spatial biases and temporal processing. To understand this link, we computationally modeled and experimentally validated the effect of laterally asymmetric delay in visual feedback on motor learning and its transfer to the control of drawing movements without visual feedback. In the behavioral experiments, we asked healthy participants to perform lateral reaching movements while adapting to delayed visual feedback in either left, right, or both workspaces. We found that the adaptation transferred to blind drawing and caused movement elongation, which is consistent with a state representation of the delay. However, the pattern of the spatial effect varied between conditions: whereas adaptation to delay in only the left workspace or in the whole workspace caused selective leftward elongation, adaptation to delay in only the right workspace caused drawing elongation in both directions. We simulated arm movements according to different models of perceptual and motor spatial asymmetry in the representation of delay and found that the best model that accounts for our results combines both perceptual and motor asymmetry between the hemispheres. These results provide direct evidence for an asymmetrical processing of delayed visual feedback that is associated with both perceptual and motor biases that are similar to those observed in hemispatial neglect syndrome.

## Introduction

When integrating external information for the execution of accurate hand movements, our sensorimotor system overcomes two challenges: laterality and time delays. Laterality is a result of processing asymmetrical visual information across space (Reuter-Lorenz et al., 1990). Time delays are a result of sensory information transmission and processing time, and they may vary between modalities (Hopfield, 1995). Previous studies investigated how the sensorimotor system compensates for differences in the spatial representations between the left and right workspaces (Heilman and Valenstein, 1979; Ziemann and Hallett, 2001; Koch et al., 2011), and for the delays between the different modalities (Miall et al., 1985; Miall and Jackson, 2006; Pressman et al., 2007; Di Luca et al., 2011; Nisky et al., 2011; Honda et al., 2012; Rohde et al., 2014; Avraham et al., 2017a; Farshchian et al., 2018). In this study, we use adaptation and transfer of adaptation paradigms to examine the interplay between these two compensatory processes.

A widely accepted view of sensorimotor control suggests that the execution of accurate movements under various environmental conditions relies on the existence of internal models (Jordan and Rumelhart, 1992; Wolpert and Miall, 1996; Wolpert, 1997; Kawato, 1999). A forward model is an internal representation of the environment that predicts the sensory consequences of a motor command and helps to compensate for changes in the sensory feedback during motor adaptation (Wolpert et al., 1995; Miall et al., 2007). In adaptation studies, the internal representation is typically evaluated from the movements of participants during and after exposure to visual or force perturbations. Throughout the adaptation, the participants modify the kinematics and dynamics of their movements to reduce errors and to maximize task success (Shadmehr and Mussa-Ivaldi, 1994; Cohn et al., 2000; Krakauer et al., 2000; Simani et al., 2007). A common way to assess the adaptation and the construction of an internal model is by examining aftereffects when the perturbation is unexpectedly removed. Another approach is to test for transfer of adaptation to a different workspace (Shadmehr and Mussa-Ivaldi, 1994; Rotella et al., 2015), a different context (Kluzik et al., 2008), or a different task (Shadmehr and Mussa-Ivaldi, 1994; Botzer and Karniel, 2013). Investigating aftereffects and transfer of adaptation reveals how the new kinematics or dynamics are represented by the motor system.

In this study, we examined adaptation to a laterally asymmetric visuomotor delay. We considered the transfer of adaptation to a 150 ms delay that was applied selectively to the visual feedback of hand movements according to the direction of the movement (and consequently, according to the workspace where the movement was applied). Meaning, participants were exposed to a lateral perturbation that was inconsistent between the two workspaces. Previously, spatially uniform visuomotor delay has been shown to cause alterations in movements' extent (Botzer and Karniel, 2013; Avraham et al., 2017a). These studies suggested that the sensorimotor system copes with delayed visual feedback by manipulating the current state variables, and specifically, by changing the gain in the internal representations. In addition, it was previously shown that the human brain has the ability to learn context-dependent perturbations, and to use spatial cues to adapt to multiple different environments (Epstein et al., 1997; Wolpert et al., 1998; Woolley et al., 2007; Howard et al., 2010; Ayala et al., 2015). Therefore, we hypothesized that when presented with an asymmetric delay that is dependent on the workspace and direction in which the target is presented, participants will form an asymmetrical state representation. We expected that this asymmetrical state representation will be demonstrated by asymmetric aftereffects and asymmetric transfer of adaptation to different tasks.

The hemispheres are different in both perceptual and motor processing, and therefore, it is possible that the hemisphere that processes the visuospatial information will also influence the effect of asymmetric delay on lateral movements. Regarding to perceptual processing, the hemispheres exhibit asymmetrical visuospatial perceptual attention, also known as “right hemisphere dominance.” The right hemisphere holds representations of both left and right fields (Heilman and Valenstein, 1979) and is able to inhibit the left hemisphere (Ziemann and Hallett, 2001; Koch et al., 2011), whereas the left hemisphere holds representations of only the right visual field. This implies that presenting delay only in one workspace, when the participant is located in the center, between the two workspaces, might be processed differently between the hemispheres. Another important aspect of lateral right-handed movements is an asymmetry in the visuomotor control of the right hand in right-handers. It is well established that the left hemisphere is involved in right-handed movements toward both right and left workspaces. However, it has also been shown that the right hemisphere can contribute to the control of movements toward the contralateral hemispace with the right hand (Farnè et al., 2003; Heilman and Valenstein, 2010). These perceptual and motor asymmetries in the hemispheres can affect lateral movements when exposed to asymmetrical visual processing across space.

To simulate the possible effects of asymmetric delay, we generated predicted arm movements according to different possible effects on transfer of adaptation with and without laterality in the temporal processing. To validate our model, we performed an experiment in which we exposed participants to direction- and workspace- specific delay between the hand and the visual cursor while performing reaching movements to both left and right targets. We examined the effect of this delay on the amplitude of the reaching movements. To probe for laterality-related changes in the internal representation due to the delay, we investigated the transfer of adaptation to a blind circle-drawing task, in which participants were requested to draw two-dimensional circles with multiple movement directions without visual feedback. We chose a blind drawing task because it allows for the detection of asymmetries in a continuum of directions (Punt et al., 2013); also, eliminating the visual feedback allows for testing the effects of adaptation to delay when participants rely only on feedforward control and proprioceptive feedback.

We found aftereffects of adaptation to delayed visual feedback in reaching movements, and transfer of adaptation to blind drawings. Interestingly, while the reach aftereffects reflected the spatial pattern of the delay perturbation, the transfer effects had significant asymmetries between delay conditions: only when the delay was presented in leftward reaches, regardless of whether it was also presented in the rightward reaches, participants exhibited asymmetrical neglect-like blind drawings. These results are only consistent with a computational model that includes perceptual and motor asymmetry which involves laterality and right hemisphere dominance.

## Methods

### Simulation of arm movement

To investigate the possible hypotheses for the effect of the asymmetric delay on participants' movements, we used a computational model. Previous studies showed an increase in movement amplitude following adaptation to visuomotor delay (Botzer and Karniel, 2013; Avraham et al., 2017a), and therefore, we simulated the hand movement following adaptation to delay with a magnifying gain in its amplitude. First, we examined the effect of delay without considering any effects of laterality. In this case, the magnifying gain was applied in the control

of movements that were performed toward the same direction in which the delay was presented. Second, we examined the effect of laterality in our experiment by testing the effect of perceptual and motor asymmetry in the hemispheres (separated and combined). Here, magnifying gain was applied following excitation of the relevant hemisphere, and inhibitory effect was reflected in multiplying the gain by a step function that canceled all excitation activity.

**Figure 1:**
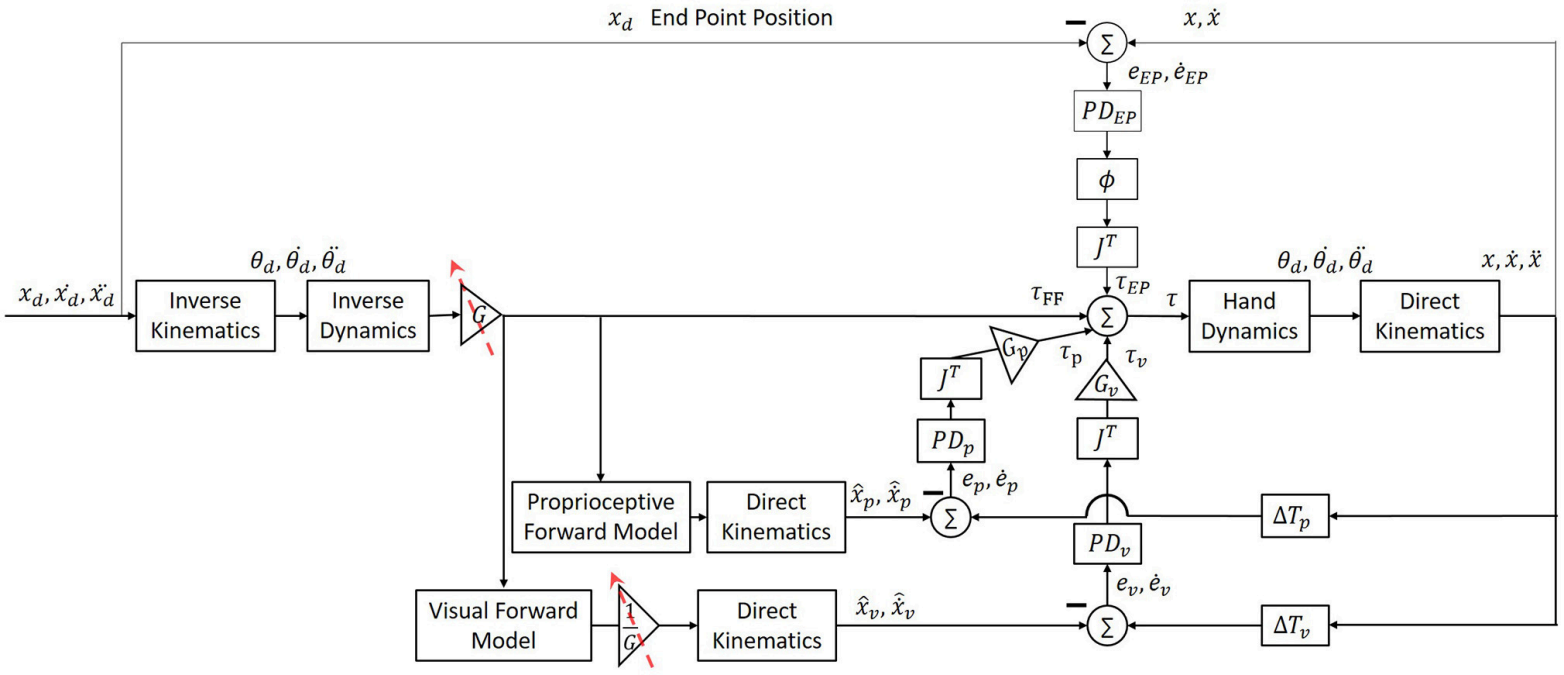
Simulation of arm movement with end-point, feedforward and two feedback controllers. The feedforward controller is an inverse model of the arm (“inverse dynamics” box) that is used to calculate the desired torques for the execution of a desired trajectory. The feedback controller calculates the torques proportional to the error between the desired and actual position and velocity. This controller includes two separate forward models and PD controllers for vision and proprioception (“Proprioceptive Forward Model” and “Visual Forward Model” box, and “*PD*_*P*_” and “*PD*_*V*_” boxes, respectively). The contribution of each modality is multiplied by the transposed Jacobian (“*J*^*T*^”) to convert to joint coordinates, and is weighted by a gain (“G_P_“ and “G_V_” boxes). An additional end-point controller is used in order to reduce the error between the actual position of the hand to the desired end point, and is weighted by a sigmoid function ∅(*t*), which increases the contribution of the end-point controller at the end of the movement. The endpoint feedback is also multiplied by the transposed Jacobean (“*J*^*T*^”) to transform to joints coordinates. Overall, the final torques are a combination of the output signals from all four controllers.

To simulate arm movements, we modeled arm dynamics as a two link model with two joints: shoulder (θ_*s*_) and elbow (θ_*e*_) (Pressman et al., 2008; Nisky et al., 2011). We simulated a simplified control of arm movement with two controllers of trajectory and end-point (Scheidt and Ghez, 2007; Botzer and Karniel, 2013), as depicted in Figure 1. The trajectory controller consisted of a feedforward controller—an inverse model of the arm, and two feedback controllers—for vision and for proprioception (Ghez et al., 2007; Scheidt and Ghez, 2007; Scheidt and Stoeckmann, 2007; Scheidt et al., 2011), and received as an input a desired trajectory. The endpoint controller was implemented as a spring and a damper with an equilibrium at the desired static end of movement, and it stabilized the arm at the end of movement. This model was used to simulate both reaching movements and blind circular movements. To simulate lateral reaching movements, we assumed that the controller tracks a planned minimum-jerk trajectory defined as a smooth trajectory from start to end-position along the x-axis (Flash and Hogan, 1985). Desired circular movements were defined by fitting a 12th order polynomial function to a desired trajectory, in order to achieve smooth velocity and acceleration along with the desired path. Note that this particular structure was chosen as an example to allow for showing the effects of different assumptions of laterality and delay interplay, and our reasoning does not depend on this particular structure or the assumption of the existence of a desired trajectory.

The desired trajectory was presented in Cartesian coordinates, and therefore we used the inverse kinematics equations with the parameters of length (*l*) of the upper and forearm in order to transform to joints space (Equation 1). The torques required to perform a desired movement were computed from Equations (2)–(5). Equation (2) depicts the dynamics of a two links arm model. Values of arm parameters of mass (*m*), length (*l*), center of mass (*l*_*c*_) and inertia (*I*) of both upper arm (shoulder) and forearm (elbow) are similar to those used in (Scheidt and Ghez, 2007). Additionally, we implemented three PD controllers for proprioceptive (Equation 3) and visual (Equation 4) feedback, and for end-point controller (Equation 5). The end-point controller contribution is weighted by a sigmoid function ∅(*t*), and both end-point and feedback controllers are multiplied by *J*^*T*^. Position and velocity error (*e* and ė) is defined as the difference between the actual to the desired arm position and velocity, respectively. The values of all proportional (K) and derivative (B) controllers are presented in Table 1. In addition, hand dynamics were simulated using (Equation 6)—the dynamics of a two-link arm. Arm parameters are as in Equations (1) and (2). To transform the desired trajectory from joint space to Cartesian space we used the direct kinematics (Equation 7).

(1) $$\left[\begin{array}{c} \theta_{s}\\ \theta_{e} \end{array}\right]\;=\left[\begin{array}{c} \;\tan^{-1}\left(x_{2},x_{1}\right)-\tan^{-1}\left(l_{e}\sqrt{1-{\left(\frac{x_{1}^{2}+x_{2}^{2}-l_{e}^{2}-l_{s}^{2}}{2l_{e}l_{s}}\right)}^{2}},l_{s}+\frac{x_{1}^{2}+x_{2}^{2}-l_{e}^{2}-l_{s}^{2}}{2l_{s}}\right)\\ \tan^{-1}(\sqrt{1-{\left(\frac{x_{1}^{2}+x_{2}^{2}-l_{e}^{2}-l_{s}^{2}}{2l_{e}l_{s}}\right)}^{2}},\frac{x_{1}^{2}+x_{2}^{2}-l_{e}^{2}-l_{s}^{2}}{2l_{e}l_{s}}) \end{array}\right]$$

(2) $$\left[\begin{array}{c} \tau_{sFF}\\ \tau_{eFF} \end{array}\right]\;=\left[\begin{array}{cc} I_{s}+I_{e}+m_{e}\left(l_{s}{}^{2}+l_{ce}{}^{2}+l_{s}l_{ce}\cos\left(\theta_{e}{}_{d}\right)\right)+m_{s}l_{cs} & I_{e}+m_{e}\left(l_{ce}{}^{2}+l_{s}l_{ce}\cos\left(\theta_{e}{}_{d}\right)\right)\\ I_{e}+m_{e}\left(l_{ce}{}^{2}+l_{s}l_{ce}\cos\left(\theta_{e}{}_{d}\right)\right) & I_{e}+m_{e}l_{ce}{}^{2} \end{array}\right]\left[\begin{array}{c} \overset{..}{\theta_{s}{}_{d}}\\ \overset{..}{\theta_{e}{}_{d}} \end{array}\right]+\left[\begin{array}{cc} -m_{e}l_{s}l_{e}\sin\left(\theta_{e}{}_{d}\right)\dot{\theta_{e}{}_{d}} & -m_{e}l_{s}l_{e}\sin\left(\theta_{e}{}_{d}\right)\left(\dot{\theta_{e}{}_{d}}+\dot{\theta_{s}{}_{d}}\right)\\ m_{e}l_{s}l_{e}\sin\left(\theta_{e}{}_{d}\right)\dot{\theta_{e}{}_{d}} & 0 \end{array}\right]\left[\begin{array}{c} \dot{\theta_{s}{}_{d}}\\ \dot{\theta_{e}{}_{d}} \end{array}\right]$$

(3) $$\tau_{p}=-G_{p}\cdot J^{T}\cdot \left(K_{p}\cdot e_{p}+B_{p}\cdot {\dot{e}}_{p}\right)$$

(4) $$\tau_{v}\;=-G_{v}\cdot J^{T}\cdot \left(K_{v}\cdot e_{v}+B_{v}\cdot {\dot{e}}_{v}\right)$$

(5) $$\tau_{EP}=-J^{T}\cdot \emptyset \left(t\right)\cdot \left(K_{EP}\cdot e_{EP}+B_{EP}\cdot {\dot{e}}_{EP}\right)$$

(6) $$\left[\begin{array}{cc} I_{s}+I_{e}+m_{e}\left(l_{s}{}^{2}+l_{ce}{}^{2}+l_{s}l_{ce}\cos\left(\theta_{e}\right)\right)+m_{s}l_{cs} & I_{e}+m_{e}\left(l_{ce}{}^{2}+l_{s}l_{ce}\cos\left(\theta_{e}\right)\right)\\ I_{e}+m_{e}\left(l_{ce}{}^{2}+l_{s}l_{ce}\cos\left(\theta_{e}\right)\right) & I_{e}+m_{e}l_{ce}{}^{2} \end{array}\right]\left[\begin{array}{c} \overset{..}{\theta_{s}}\\ \overset{..}{\theta_{e}} \end{array}\right]+\;\left[\begin{array}{cc} -m_{e}l_{s}l_{e}\sin\left(\theta_{e}\right)\dot{\theta_{e}} & -m_{e}l_{s}l_{e}\sin\left(\theta_{e}\right)\left(\dot{\theta_{e}}+\dot{\theta_{e}}\right)\\ m_{e}l_{s}l_{e}\sin\left(\theta_{e}\right)\dot{\theta_{e}} & 0 \end{array}\right]\left[\begin{array}{c} \dot{\theta_{s}}\\ \dot{\theta_{e}} \end{array}\right]=\left[\begin{array}{c} \tau_{s}{}_{H}\\ \tau_{e}{}_{H} \end{array}\right]$$

(7) $$\left[\begin{array}{c} x_{1}\\ x_{2} \end{array}\right]\;=\left[\begin{array}{c} l_{e}\cdot \cos\left(\theta_{s}+\theta_{e}\right)+l_{s}\cdot \cos(\theta_{s})\\ l_{e}\cdot \sin\left(\theta_{s}+\theta_{e}\right)+l_{s}\cdot \sin(\theta_{s}) \end{array}\right]$$

**Table 1:** Values of proportional and derivative controllers.

**Parameter**	**Value**
K_p_	0.9 N/m
B_p_	0.7 N·s/m
K_v_	0.9 N/m
B_v_	0.7 N·s/m
K_EP_	0.9 N/m
B_EP_	0.7 N·s/m
Visual Gain (Gv)	0.7
Proprioception Gain (Gp)	0.3
Adaptation Gain (G)	1.2

*These values were consistent for all simulations*.

The predicted arm position and velocity were computed from the inverse controller torques with a forward model. We also assumed that any changes in the inverse model as a result of adaptation will also lead to changes in the visual forward model. By using two different forward models and feedback controllers for vision and proprioception, we were able to differentiate between movements with and without visual feedback. The end-point controller did not change throughout the simulation, and was always used to stabilize the hand at the desired end-position. This controller was multiplied by a sigmoid function $\emptyset \left(t\right)=\frac{1}{1+e^{-a\left(t-c\right)}}$, which increased the contribution of the end-point controller according to a desired timing along the movement (by choosing the value of c). Before adapting to the delay, the time when the sigmoid function was equal to 0.5 was at the end of movement. After adapting to the delay, we assumed that as a result of uncertainty during the movement, the end-point controller will be tuned earlier—approximately in the middle of the movement.

The different stages in the experiment (pre-exposure, early-adaptation, late-adaptation, and post-exposure) were simulated by changing the visual delay and the magnifying gain that represented the delay in the sensorimotor system. In all simulations, we considered the intrinsic visual and proprioceptive delay as no delay, as they are present in all conditions of the experiment. Before the exposure to delay (pre-exposure), the visual feedback was not altered (ΔT_v_ = 0) and no adaptation process has occurred yet (G = 1). At early exposure before adaptation has occurred (early-adaptation), the visual delay was set to ΔT_v_ = 150 ms and the gain still did not change (G = 1). After adapting to the delay (late-adaptation), the gain was changed to G = 1.2 such that the desired trajectory was extended in the direction of the movement. In this stage, the visual delay was ΔT_v_ = 150 ms. To simulate the removal of the delay in the post-exposure stage and the aftereffects, the visual delay was changed to ΔT_v_ = 0, and the gain in this stage was still G = 1.2. Throughout the experiment, the proprioceptive delay was not changed, and therefore we set ΔT_p_ = 0.

### Participants and experimental setup

Sixty-five right-handed healthy volunteers (ages 18–35, 38 females and 27 males) participated in the study after signing the informed consent form as approved by the Human Participants Research Committee of Ben-Gurion University of the Negev, Be'er-Sheva, Israel. The participants were all naive to the purpose of the experiment and were reimbursed for their participation.

The experiment was administered in a virtual reality environment in which the participant held a robotic arm: six degrees of freedom (DOF) PHANTOM® Premium™ 1.5 haptic device (Geomagic®), controlled by a dedicated C++ code. Participants held the robotic arm with their right hand controlling a cursor displayed on a screen and aligned with their hand location, with a delay of 10 ms because of the display control rate. Participants' hand was hidden from sight the entire experiment by the screen that was located horizontally above their hand, and by a sheet that covered their upper body. Hand movements were constrained to the horizontal plane by an air sled wrist-supporter that reduces friction with the surface.

### Protocol

**Figure 2:**
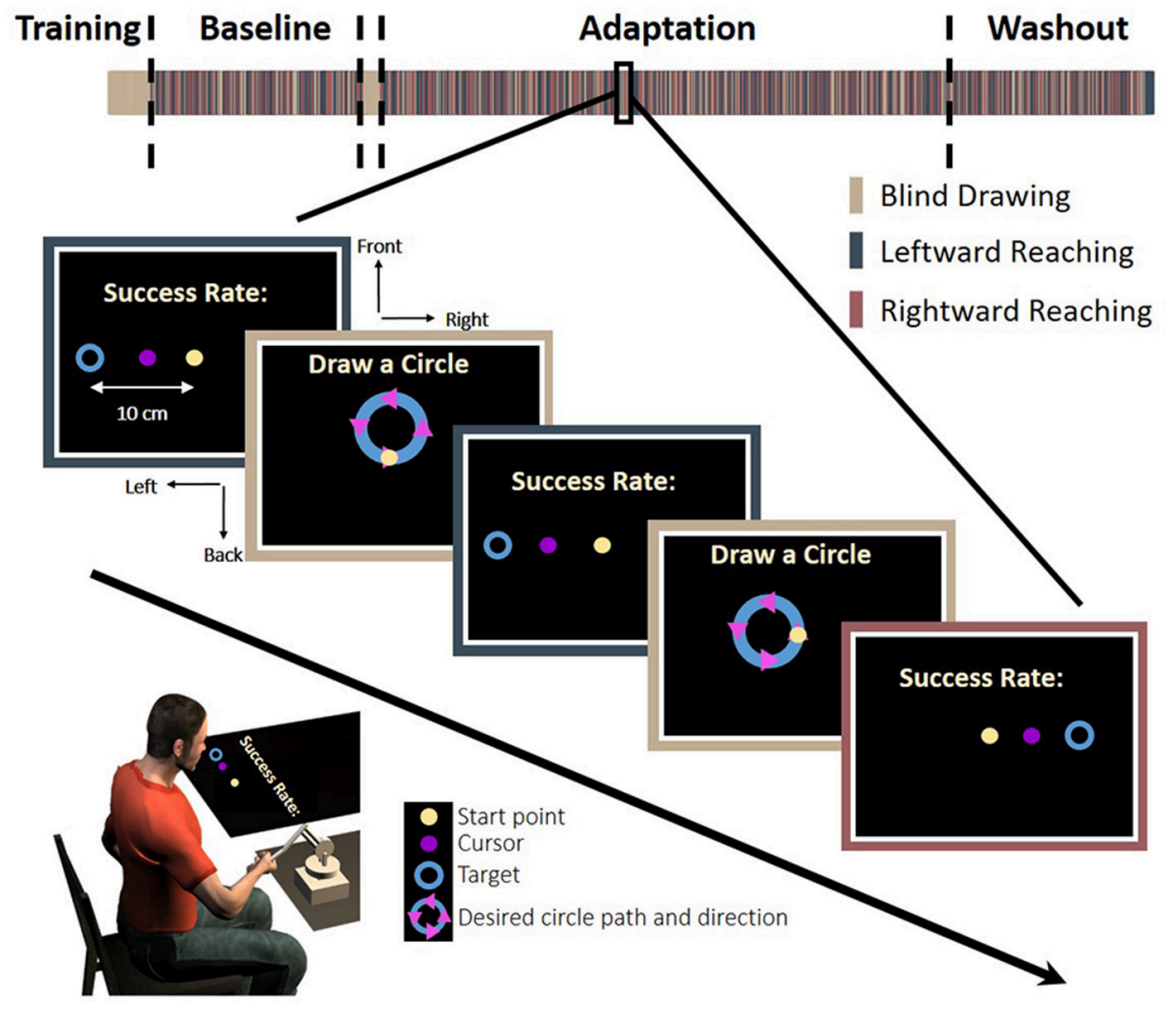
Experimental protocol. In each trial, participants were required to make a *reaching*: move a cursor between a start and an end target to the left (blue bar) or the right (red bar), or to make a *blind drawing*: draw a circle without visual feedback (beige bar). In the reaching, a start point (light yellow), a target (blue circle), and a cursor were presented. To motivate the participants, we presented a success rate representing the percentage of accurate trials—the trials in which the participants hit the target—out of all reaching trials in the experiment until that time. In the blind drawing, a desired circle path (blue) was presented together with arrows that indicated movement direction (magenta triangles), but no cursor was presented. Overall, there were eight different kinds of circular movements: four different locations—front, back, right, and left, and two different directions—clockwise and counterclockwise. The experiment was divided into three sessions: Baseline, Adaptation, and Washout. During the Baseline and Washout sessions, the cursor movement in the reaching task was concurrent with the movement of the hand. During the Adaptation session, the visual feedback was delayed by 150 ms in movements toward the leftward, the rightward, or both targets (see section Methods for details).

The experiment consisted of two tasks: reaching movements to left or right targets and circle drawing without visual feedback. The trials were presented in a random predetermined order. In the reaching task, a trial was initiated when participants placed a circular cursor, 1 cm diameter, inside a starting point with the same size. The task was to move the cursor from the starting point to a circular target, 2 cm diameter, which appeared in the left or the right side of the task space, at a distance of 10 cm away from the starting position (Figure 2).

Movement started when the color of the cursor changed after a fixed period of time in which the participant was stationary at the start position; this instructed the participant to perform a smooth point-to-point center-out reaching movement. Movement ended when the velocity was less than 1 cm/s. Following the movement, the visual feedback was turned off and the robot applied a spring-like force that returned the hand to the start position. Due to the nature of our temporal perturbation, we wished to assure similar movement speeds, and therefore, the participants received a feedback about the velocity of their movement. When the maximum velocity was lower than 30 cm/s, the word “Faster” appeared on the screen, and when the velocity was higher than 50 cm/s, the word “Slower” was displayed. To motivate the participants to make accurate movements to the target, they received feedback about the accuracy of their movement. Accurate movements were defined as those in which the center of the cursor was in the range of ± 1 cm from the center of the target. To provide a feedback about the end movement position, we presented the location of the cursor with a color cue that indicated the accuracy of the movement (green for accurate movement and red for inaccurate movement) after 0.2 s from movement ending. In addition, we presented a success rate corresponding to the percentage of successful trials from all reaching trials in the experiment until that time.

In the circle drawing task, a circle with a radius of 3.5 cm was displayed on the screen in four different locations: front, back, right and left. Arrows on the circle indicated the direction of the drawing to either clockwise or counterclockwise. The location of the starting point was always in the middle of the task space in all conditions, identically to the location of the start point in the reaching task. A trial was initiated when participants placed a circular cursor, 1 cm diameter, inside the starting point for a fixed duration. Afterwards, the cursor disappeared and the start point changed its color, instructing the participants to initiate a smooth circular movement along the desired circle from the starting point, in the direction of the arrows. Circular movements did not have velocity constrains. The movement ended when the velocity was less than 0.5 cm/s.

Participants were assigned to one of four groups according to the workspace where they were exposed to delay: (1) only in leftward reaching movements (Left Delay, *N* = 15), (2) only in rightward reaching movements (Right Delay, *N* = 15), (3) in both leftward and rightward movements (Both Delay, *N* = 20), and (4) a control group that was not exposed to any perturbation throughout the entire experiment (No Delay, *N* = 15).

The first block of the experiment (40 trials) was training for the circle drawing task. In these training trials, participants drew the circles without visual feedback. After each trial, the drawn circle was displayed along with the desired circle and the start point. The purpose of these trials was to acquaint the participants with the task and to train them to draw circles according to a desired trajectory when no visual feedback is presented. The data from the training trials were not included in data analysis. Then, the experiment was divided into three sessions: Baseline, Adaptation, and Washout. In the Baseline session (160 reaching movements and 40 circle movements), participants performed reaching without any perturbation and with interleaved blind circle-drawings. After the baseline session, we presented participants with another block of training for the circle drawing task (16 trials). The purpose of this block was to verify that the circles drawn in the Adaptation session originated from the exposure to the applied perturbation and not from forgetting how to draw the blind circles. In the adaptation session (416 reaching movements and 104 circle movements), the visual feedback between the hand and the cursor in the reaching task was delayed by 150 ms either when the left target appeared (Left Delay, LD), when the right target appeared (Right Delay, RD), or when both right and left targets appeared (Both Delay, BD), depending on the experimental group. For the No-Delay group (ND), there was no change in the Adaptation session. During Washout (160 reaching movements and 40 circle movements), the delay was unexpectedly removed, which enabled us to examine the aftereffect of adaptation. The entire experiment lasted approximately 90 min with four breaks of 1.5 min every 160 reaching trials.

### Data analysis

Position and velocity were recorded during the entire experiment at 200 Hz and were analyzed off-line using custom-written Matlab® code (The MathWorks, Inc., Natick, MA, USA). Both position and velocity were filtered by low pass Butterworth filter with a cutoff frequency of 10 Hz [Matlab function filtfilt()]. In addition, the position was interpolated to fit the number of samples using Matlab function interpft(), which resulted in different sampling rate for each signal that depended on the number of samples in the original signal. For the purpose of data analysis, we defined reach movement initiation when the velocity rose above 5% of the maximum velocity, and movement ending when the velocity decreased below 5% of the maximum velocity. We examined the trajectory in each direction separately, by measuring the amplitude of the movement as the maximum displacement.

In the circle drawing task, due to the importance of the drawing's direction in our study, we first removed all circles that were mistakenly drawn in the direction that was opposite to the instructed direction (1.65% of all circles). Then, we defined the initiation and end of the movement by using both position and velocity. Initially, we found the locations where the hand first leaves and returns to the start position area. This was done to account for only one circle in cases when the participants drew more than one complete circle. Afterwards, we defined the actual initiation and end of the movement based on the velocity thresholds as we defined in the reaching movements. To calculate the deviation of the drawn circles from the desired circle, we measured the peak amplitude (maximum distance) of hand movement in the x and y directions.

In the analysis of the drawn circles, we did not include the data from the early-adaptation stage. From the results of the reaching task in all the conditions, we saw that participants adapted to the perturbation quite fast. Therefore, we could not verify that all drawn circles in all 8 conditions, used for the analysis, were performed in this phase of post-exposure and early-adaptation.

### Statistical analysis

The effect of the perturbation in each condition on the reaching movements was assessed by using a two-way repeated measures ANOVA with between factors of Stage (Late-Baseline/Early-Adaptation/Late-Adaptation/Early-Washout) and Direction (Leftward Movements/Rightward Movements). For the blind drawings, we initially examined the effect of delay on left and right error separately, using one-way repeated measures ANOVA with factor Stage (LB/LA/EW). After dividing between the circles according to initiation workspace, the lateral effect on the blind drawings was examined using two-way repeated measures ANOVA with within factors of Stage (LB/LA/EW) and Initiation-workspace (Left/Right). Then, we examined the differences in the Late Adaptation stage between the experiments using two-way repeated measures ANOVA with between factor of Experiment (LD/RD/BD/ND) and within factor of Initiation-workspace (Left/Right). Data were tested for normality distribution using Lilliefors test. Additionally, we used Mauchly's test to examine whether we can assume sphericity of the data. In case the sphericity assumption was not met, we used Greenhouse-Geisser adjustment. When found significant effects, *post-hoc t*-test was performed with the Bonferroni correction. Significant effects were defined at the *p* < 0.05 probability level.

## Results

### Simulation study

**Figure 3:**
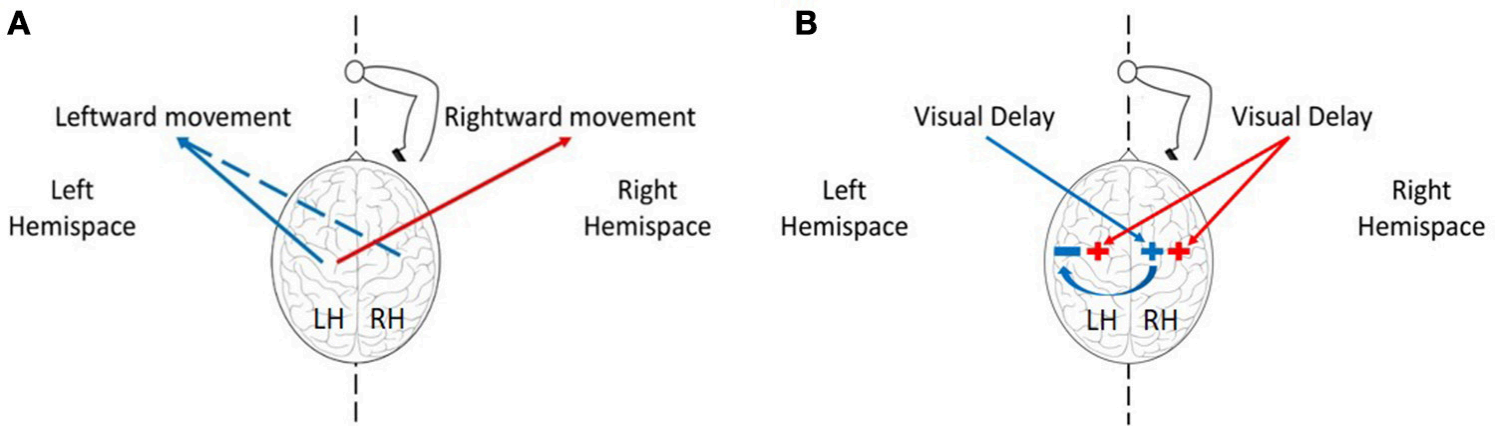
The effect of delay in left, right, or both hemispaces on rightward and leftward movements. **(A)** The effect of the hemispheres on movement extent toward both hemispaces. The left hemisphere controls movements of the right hand toward left (blue) and right (red) sides, and the right hemisphere can mediate leftward movements (dashed blue). **(B)** The effect of delayed visual feedback on the hemispheres according to the visual fields. Delay in left visual field (blue) affects motor circuits responsible for movement extension in the right hemisphere, and delay in the right visual field (red) affects both hemispheres. Following excitation of the right hemisphere after exposure to left delay, the right hemisphere inhibits motor circuits in the left hemisphere, thereby canceling any deviation toward the right hemispace after exposure to left delay (blue arrow).

Using a computational model (Figure 1), we simulated the possible effects of exposure to delay, adaptation, and transfer to blind circle drawing. To validate our simulation and to choose the different parameters, we used the previously observed effect of delay on reaching movements (Botzer and Karniel, 2013). We simulated the effect of asymmetrical delay on the lateral reaching movements before and after adaptation has occurred. Then we used the obtained simulation to examine different hypotheses for the effect of delay on transfer to vision-omitted circular movements according to motor- and perceptual-based models of hemispheric asymmetry (Figure 3).

**Figure 4:**
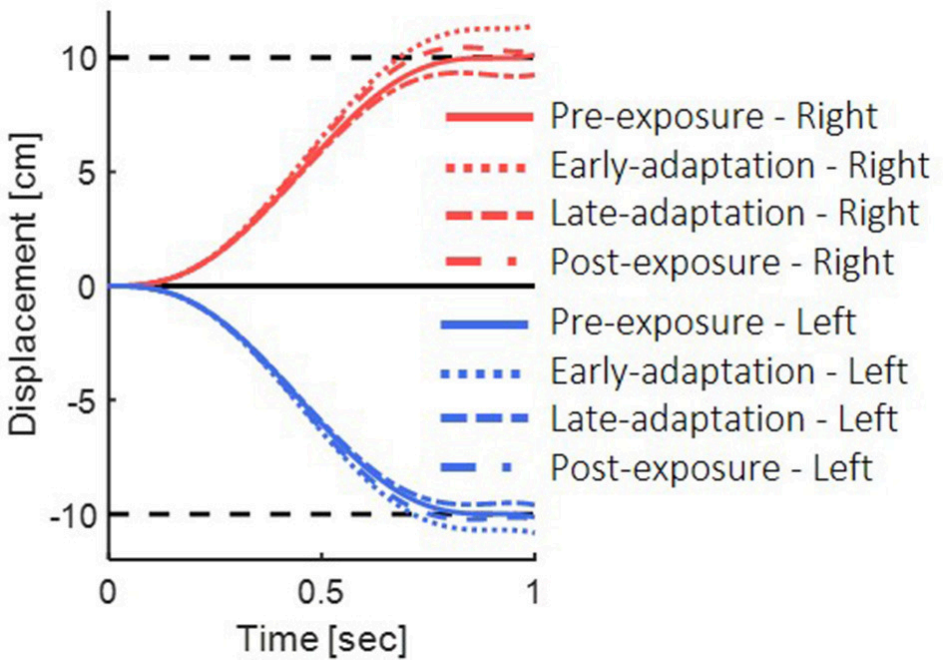
Simulation results for reaching movements with the presence of delayed visual feedback. We simulated the movements in the different phases of pre-exposure, early-adaptation, late-adaptation and post-exposure. Positive displacement indicates a rightward movement. The simulation demonstrate overshoot of the target when initially exposed to delay and undershoot when the delay is removed.

In the pre-exposure phase, no perturbation was applied, and the simulated arm followed the desired trajectory properly (Figure 4, solid lines). Before adaptation took place, the visual feedback was delayed, but no change in the feedforward or feedback controllers has occurred yet. Hence, a misalignment between the estimated location and the actual observed location of the hand during the reaching task resulted in a positive error, and the feedback controller of the visual modality caused target over-reaching (Figure 4, dotted lines).

After adapting to the delay, movement overshoots gradually decreased. We simulated adaptation to delay based on the use of gain representation (Avraham et al., 2017a). For the late-adaptation condition, we used magnifying gain (G > 1), multiplied by the output of the forward model. Meaning, the desired trajectory was extended in the direction of the movement. The visual forward model was multiplied by the inverse gain, causing a reduction of the error in the visual feedback controller, and leading to a reduction of the over-reaching pattern (Figure 4 dashed line). Following abrupt removal of the delay, the forward and inverse models were still tuned to the delayed condition. However, the visual feedback matched the real location of the hand, which resulted in negative error of the visual modality and under reaching of the target (Figure 4 dashed and dotted line).

**Figure 5:**
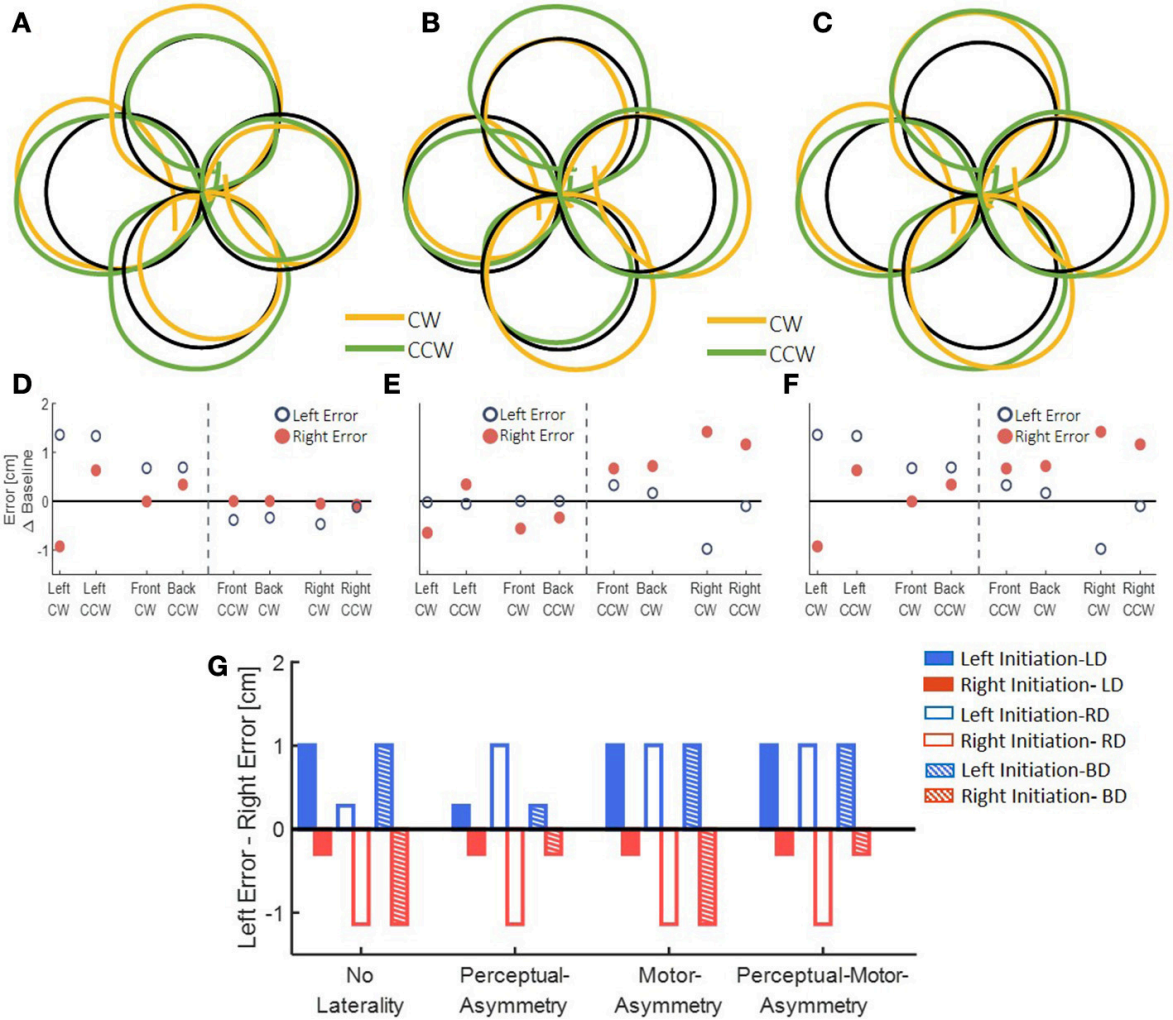
Simulation results for transfer movements after adaptation to asymmetric delay using different predictions. **(A–C)** Simulated blind circles after adaptation to delay for the *No-Laterality* model in clockwise (orange) and counterclockwise (green) directions. The circles are elongated toward the hemispace where the delay was applied. **(D–F)** Left and right error as a function of the location (front, back, right, and left) and the direction (clockwise—CW—and counterclockwise—CCW) of the drawn circle for the *No-Laterality* model. The dashed line divides the circles to left- and right-initiated circles. Both left- and right-initiated circles are elongated in the side where the delay was applied. **(G)** Summary of all possible effects for asymmetric delay. For each condition of delayed side, we expect movements to be elongated toward either the left or the right hemispaces, according to the modeled mechanism.

After simulating the reaching movements without laterality, we simulated the different models for the effect of asymmetric delay on transfer circular movements (Figure 5). First, we simulated the transfer of adaptation without any effects of laterality. This resulted in elongation of the circles toward the side where the delay was applied (Figures 5A–F). Then, we inserted laterality effects of perceptual, motor, and both perceptual and motor asymmetries (Figure 5G, Table 2). Considering only perceptual asymmetry, the gain in movement amplitude was applied when motor circuits in the left hemisphere were excited. In this case, excitation of the right hemisphere could affect the applied movements by inhibiting the activity of right hemisphere on the left hemisphere. Therefore, an elongation of the circles was only observed in the case of delay in rightward movements, for both left- and right-initiated circles. For motor asymmetry, when motor circuits in the left hemisphere were excited, the magnifying gain was uniform, causing elongation of both left- and right-initiated movements. In contrast, when motor circuits in the right hemisphere were excited, the gain was only applied in the leftward movements, and only they were elongated. This asymmetry yielded an elongation of both left- and right initiated circles in two conditions: delay in only the right workspace, and delay in both left and right workspaces. Delay in the left workspace resulted in only leftward elongation. Applying both perceptual and motor asymmetry resulted in elongation of both sides of the circles in the cases of delay in the right workspace, and only leftward elongation when the delay was in the left workspace or in both workspaces. The authors will be happy to share the code for the simulation with the interested reader.

**Table 2:** Summary of all possible effects for asymmetric delay.

**Experimental group**	**LD**		**RD**		**BD**	
**Elongated workspace**	**L**	**R**	**L**	**R**	**L**	**R**
No laterality	+	–	–	+	+	+
Perceptual asymmetry	–	–	+	+	–	–
Motor asymmetry	+	–	+	+	+	+
Perceptual-motor asymmetry	+	–	+	+	+	–

*Simulating the different mechanisms for the effect of asymmetric visuomotor delay enabled us to better understand the observed effect of adaptation*.

In the next step, by using the results of the behavioral experiment, we were able to reject some of the hypothesized models for the transfer effect.

### Behavioral experiment

#### Reaching movements-adaptation to delay affects reaching movements toward the delayed workspace

**Figure 6:**
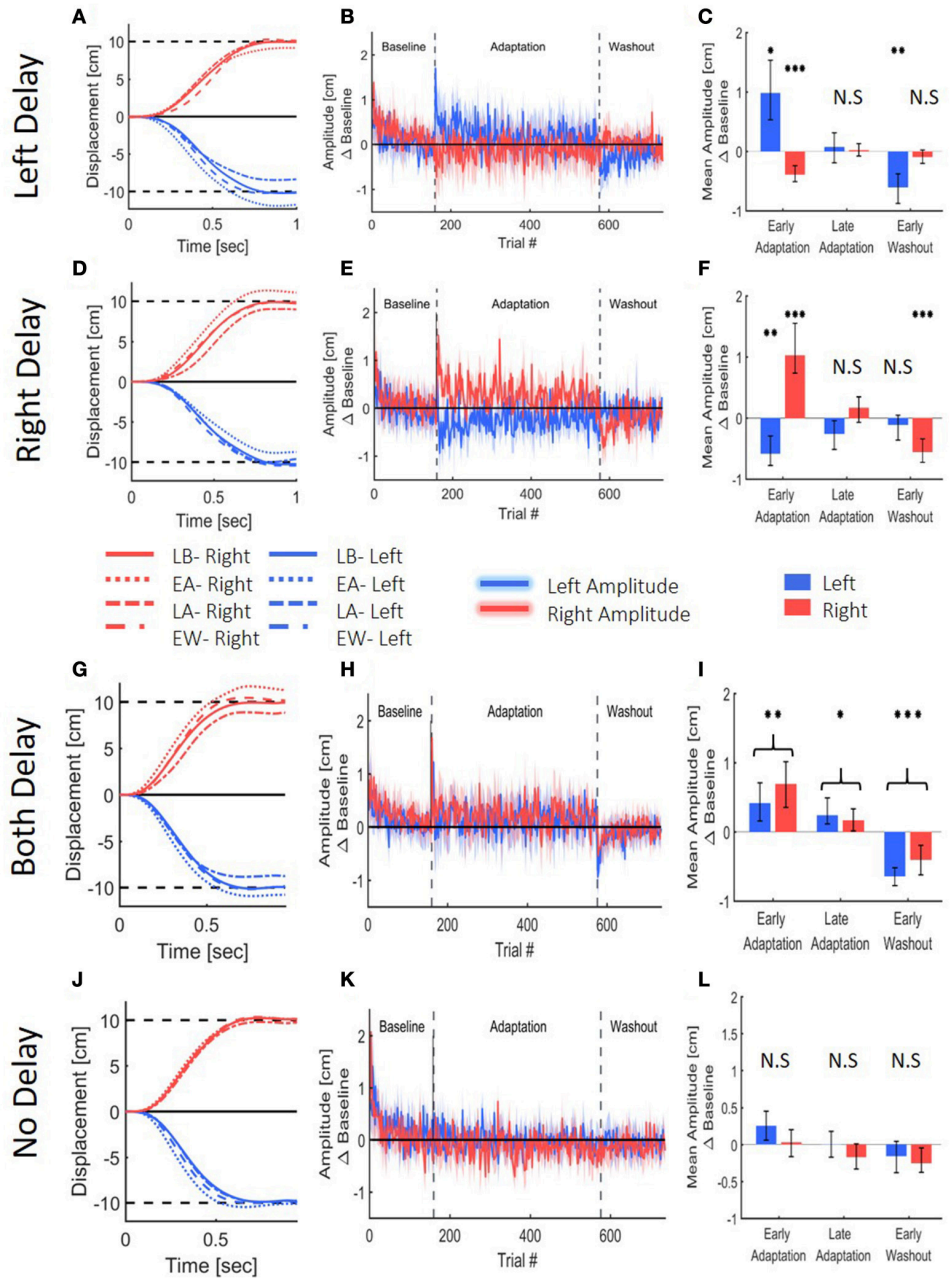
Reaching movements from the Left Delay (LD, **A–C**), Right Delay (RD, **D–F**), Both Delay (BD, **G–I**) and No Delay (ND, **J–L**) conditions. **(A)** Examples of movements of a typical participant in the Left Delay group from the Late Baseline (LB), Early adaptation (EA), Late adaptation (LA), and Early Washout (EW) stages. Positive displacement indicates a rightward movement. The participants overshoot the left target when initially exposed to delay, but they quickly adapt and restore baseline movements, and exhibit undershoot in the washout. Interestingly, the movements in the other direction are initially affected, but no aftereffects are observed. **(B)** Amplitude (line) and 95% confidence intervals (shaded region) of the leftward and rightward movements from the Left Delay condition. Results are presented after subtraction of the movement amplitude at the end of the baseline session and taking absolute value. Positive (negative) value indicates overshoot (undershoot) in the direction of movement. Leftward movements demonstrate typical pattern of adaptation, and the rightward movements exhibit an initial undershoot that is reduced with adaptation and no aftereffect. **(C)** Mean Amplitude in the presence of left delay in the first and last five movements of the Adaptation stage and the first five movements of the washout for all participants. Asterisks represents significant difference from zero: ^*^p < 0.05, ^**^p < 0.01, ^***^p < 0.001. **(D–F)** Similar but mirror results were observed in the Right Delay condition. **(G–I)** Results for the No Delay condition. Graphs and colors are as in **(A–C)**. Here, The participants overshoot both targets when initially exposed to delay, but they quickly adapt and restore baseline movements, and exhibit undershoot in the washout. **(J–L)** Results for the No Delay condition. Graphs and colors are as in **(A–C)**. No spatial deviation is observed, as expected.

To assess adaptation to delay, we first examined the extent of the lateral reaching movement. Reaching movement analysis of the left, right and both delay groups suggest that all groups adapted to the delay (Figure 6). Upon early exposure to the delay, participants over-reached the target in the workspace where the delay was applied. With repeated exposure to the perturbation, they adjusted their movements, and by the end of adaptation, they restored baseline performance. For the two groups that were exposed to asymmetrical delay (LD and RD), the participants also initially started to under-reach the target in the opposite direction, but this effect was weaker and vanished quickly. After the delay was removed, we observed an aftereffect of target under-reach only in movements toward the delayed workspace.

These observations were supported by a statistical analysis. We divided the experiment to four stages of Late Baseline (LB, 5 last movement before exposure to delay), Early Adaptation (EA, 5 first movements with the presence of delay), Late Adaptation (LA, 5 last movements with the presence of delay) and Early Washout (EW, 5 first movements after removing the delay). Within each experimental group that was exposed to asymmetric delay groups (LD and RD), we found significant changes in the movement amplitude between the different stages in the experiment, and these changes were different between left and right movements [Stage-Workspace interaction effects—LD: *F*_(0.87, 12.15)_ = 95.14, *p* < 0.001; RD: *F*_(3, 42)_ = 45.92, *p* < 0.001]. In the leftward reaches of the left delay group, we observed a typical adaptation pattern: overshoot in EA [*t*_(14)_ = 3.59, *p* < 0.05]; no difference in LA [*t*_(14)_ = 0.48, *p* = 1]; and undershoot in EW [*t*_(14)_ = 4.53, *p* < 0.01] (all with respect to LB, Figure 6C). The rightward reaches of this group exhibited a different pattern: undershoot in EA [*t*_(14)_ = 5.92, *p* < 0.001]; and no difference in LA [*t*_(14)_ = 0.27, *p* = 1] and EW [*t*_(14)_ = 1.53, *p* = 0.88]. A similar but opposite pattern was observed in the right delay group [rightward reaches: EA: *t*_(14)_ = 5.22, *p* < 0.001; LA: *t*_(14)_ = 1.57, *p* = 0.83; EW: *t*_(14)_ = 5.47, *p* < 0.001; leftward reaches: EA: *t*_(14)_ = 4.83, *p* < 0.001, LA: *t*_(14)_ = 2.01, *p* = 0.38, and EW: *t*_(14)_ = 1.14, *p* = 1, Figure 6F]. Overall, in both the left and right delay groups, the participants adapted to the asymmetric visuomotor delay by adjusting their movement amplitude selectively in the workspace where the delay was applied, and exhibited significant aftereffects of adaptation. The initial undershoot to the other workspace during early exposure to the delay quickly vanished, and there were no aftereffects in the non-delayed workspace.

The extent of reaching movements for the both delay group demonstrated a typical pattern of adaptation that was similar in both directions (Figures 6G–I). There was a statistically significant difference in movement extent between the stages [Stage- *F*_(1.55, 29.26)_ = 60.51, *p* < 0.001], but no difference between leftward and rightward movements in the different stages [Direction- *F*_(0.51, 9.75)_ = 2.78, *p* = 0.13 and Direction-Stage interaction- *F*_(1.55, 29.26)_ = 2.38, *p* = 0.12]. When the delay was first introduced, movements over-reached the target in both sides [*t*_(19)_ = 4.27, *p* < 0.01]. Continued exposure to delay in both workspaces led to a reduction of the over-reaching pattern, though the adaptation was not fully achieved compared to baseline performances [*t*_(19)_ = 3.11, *p* < 0.05]. When the delay was removed, participants under-reached the target in both sides [*t*_(19)_ = 7.74, *p* < 0.001]. These results indicate that when the visual feedback is delayed in both workspaces, the participants adapted to the perturbed visual feedback, and exhibited aftereffects in both workspaces.

The control group did not experience any visual perturbation (Figures 6J–L), and did not demonstrate any deviation in movement extent. This corroborates our claim that the observed spatial deviations are a result of the delayed visual feedback.

#### Blind drawing task-transfer of adaptation causes spatial asymmetry that depends on the delayed workspace

To test the transfer of adaptation, we examined the symmetry of blind circle drawing movements that were interleaved with reaching movements. To assess the symmetry, we calculated the left and right error by measuring the maximum deviation in each direction relatively to the ideal circle (that was presented on the screen).

**Figure 7:**
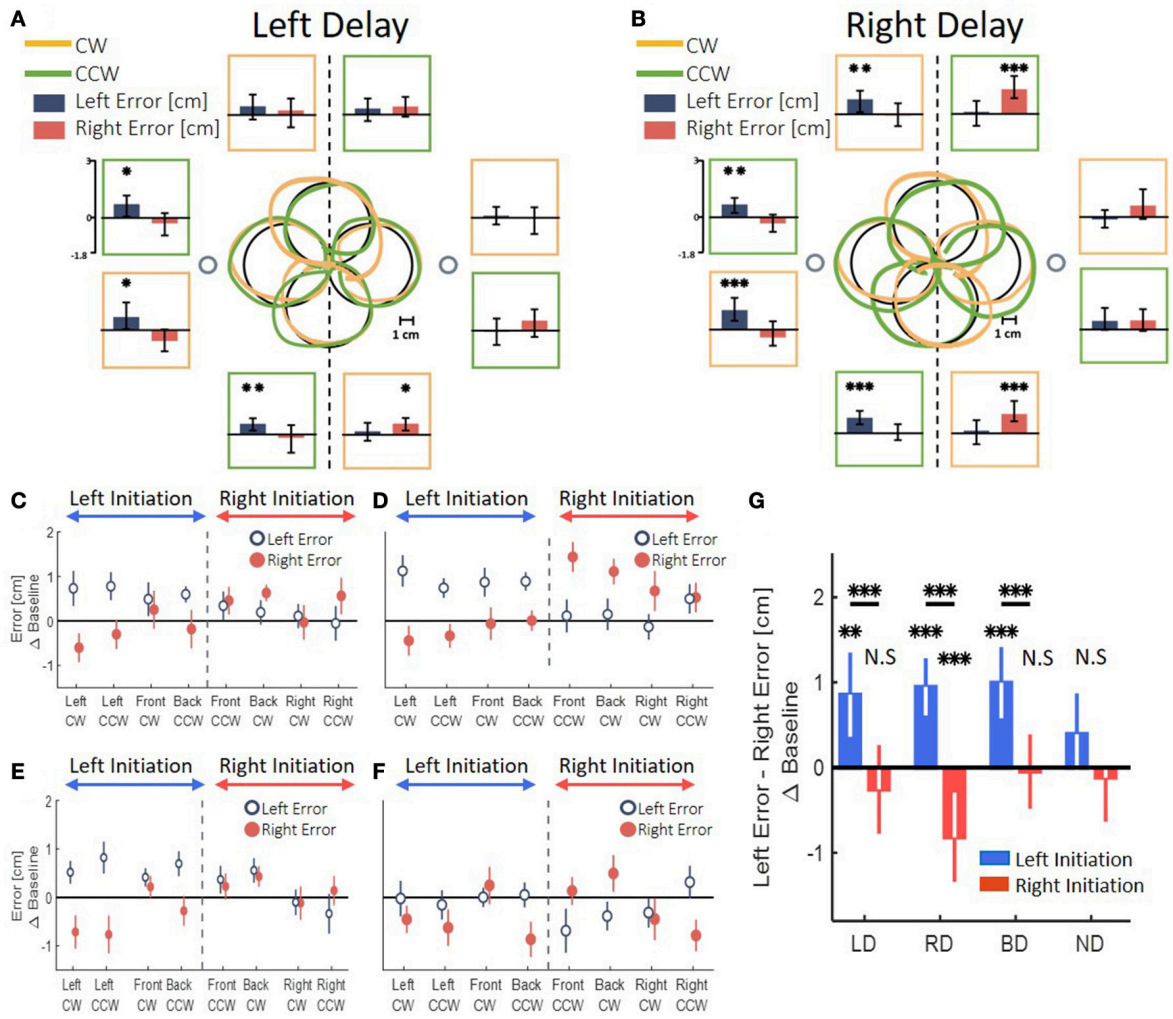
Left and right deviation from the desired trajectory in the circle drawing task. **(A)** At the center, examples of individual movements of a typical subject that illustrate the deviation of the drawn circles, for both clockwise (orange) and counterclockwise (green) circles. Large black circles are the ideal drawings, and the two small circles are the targets from the reaching task (drawn at scale). Panels around the center present mean of left (dark blue) and right (dark red) error for circles drawn in the end of adaptation session in the presence of delay only in the left side of the tasks space. The panels are located spatially to represent the location and drawing direction of the circles. Asterisks represents significant difference from zero: ^*^p < 0.05, ^**^p < 0.01, ^***^p < 0.001. **(B)** Similar to **(A)** following adaptation to a delay only in the right side of the tasks space. Asterisks are as in **(A)**. **(C)** Left and right error following adaptation to Left Delay as a function of the location (front, back, right and left) and the direction (clockwise—CW—and counterclockwise—CCW) of the drawn circle. The dashed line divides the circles to left- and right-initiated circles. The elongation is observed only in the left side of the left-initiated circles. **(D)** Left and right error following adaptation to Right Delay. Surprisingly, the result of the Right Delay is not a mirror picture of the Left Delay condition. Instead, both left- and right-initiated circles are elongated in the side of their initiation hemispace. **(E)** Both delay condition. The error is different according to the side where the drawing is initiated: when the drawing is initiated in the left–left error is larger than right error, and when the circles are initiated in the right–no deviation is observed. **(F)** No Delay condition. No similar pattern of difference between left and right error is observed. **(G)** Statistical analysis of the difference in left and right error for all groups in the experiment. Asterisks are as in **(A)**. Left and Both Delay groups show deviation only toward the left side. Right Delay group shows deviation to both sides.

In all the groups that were exposed to the delay, the transfer of adaptation yielded a clear spatial elongation in the blindly drawn circles. However, the pattern of elongation was distinct between the different delay conditions. In a striking contrast to the effects of left and right delay on the reaching movements, the patterns of elongation differed substantially between the asymmetric delay groups LD and RD in the circle drawing task. An example of drawings following adaptation to left delay is depicted in Figure 7A. By examining the left and right errors for each circle (Figure 7A, dark blue and light red bars), we saw that following adaptation to left delay, the circles that started in the left workspace (left-initiated circles) were elongated to the left, whereas the circles that started in the right workspace (right-initiated circles) were not elongated at all. In contrast, following adaptation to right delay, participants drew both left- and right-initiated circles that were elongated to the direction of their initiation; i.e., left-initiated circles were elongated to the left, and right-initiated circles were elongated to the right (Figure 7B). The effect of the initiation workspace is especially highlighted in the front and back circles: the side of the elongation is determined by the clockwise (CW) and counterclockwise (CCW) drawing direction (orange and green traces, respectively) rather than by the spatial location of the circle.

We divided between the circles according to their initiation workspace—left-initiated circle are: left, front CW and back CCW, and right-initiated circles are: right, front CCW and back CW (Figures 7C,D). Applying similar analysis for the both-delay group, revealed that transfer of adaptation to the blind drawing task resulted in a striking resemblance to those of the left-delay group, showing only elongation of left-initiated circles (Figure 7E). In the control experiment, with no perturbation (Figure 7F), the circles were nearly symmetrical without any lateral pattern. This corroborates that the elongation of the blind circles is not caused by unrelated effects of our setup or fatigue.

The transfer effect of delay on the blind circular drawing movements persisted also in the washout stage. This was despite the fact that the extent of the reaching movements returned very quickly to those observed in the Baseline.

To highlight the laterality in the spatial effects, we performed a summarizing analysis. In this analysis, we distinguished between the circles based on the workspace of the initial drawing movement. Then, we calculated the difference between the left and right errors for each group (Figure 7G). In the LD group, we found a significant change in the elongation of the circles between the stages [Workspace-Stage interaction effect: *F*_(0.97, 57.23)_ = 18.14, *p* < 0.001]. Specifically, the left errors were significantly larger than the right errors (meaning left elongation) only for the left-initiated circles during both Late Adaptation [LA: *t*_(59)_ = 3.47, *p* < 0.01] and Washout [W: *t*_(59)_ = 3.96, *p* < 0.001]. In the RD group, we also found a significant change in the elongation of the circles between the stages [Workspace-Stage interaction effect: *F*_(0.96, 57)_ = 76.44, *p* < 0.001]. However, following adaptation to right delay, the left errors were significantly larger than the right errors in left-initiated circles [LA: *t*_(59)_ = 6.74, *p* < 0.001; W: *t*_(59)_ = 4.83, *p* < 0.001] and right errors were significantly larger than the left errors (meaning right-elongation) in right-initiated circles [LA: *t*_(59)_ = 3.29, *p* < 0.01; W: *t*_(59)_ = 4.17, *p* < 0.001]. For the BD group, we found a significant main effect of initiation workspace, stage, and the interaction between stage and initiation workspace [*F*_(0.54, 42.75)_ = 226.45, *p* < 0.001, *F*_(1.1, 85.5)_ = 7.8, *p* < 0.01, and *F*_(1.1, 85.5)_ = 11.68, *p* < 0.001, respectively]. Even though the delay perturbation was presented in both sides, only the left-initiated circles were elongated to the left [Figure 7G, positive difference between left and right error compared to the baseline difference LA: *t*_(79)_ = 4.75, *p* < 0.001; W: *t*_(79)_ = 3.65, *p* < 0.01], and the right-initiated circles were not elongated at all [LA: *t*_(79)_ = 0.23, *p* = 1; W: *t*_(79)_ = 0.25, *p* = 1]. The comparison of this elongation pattern with the simulation results (Figure 5G) suggests that the effects are caused by a perceptual-motor asymmetry in the processing of the delayed feedback.

**Figure 8:**
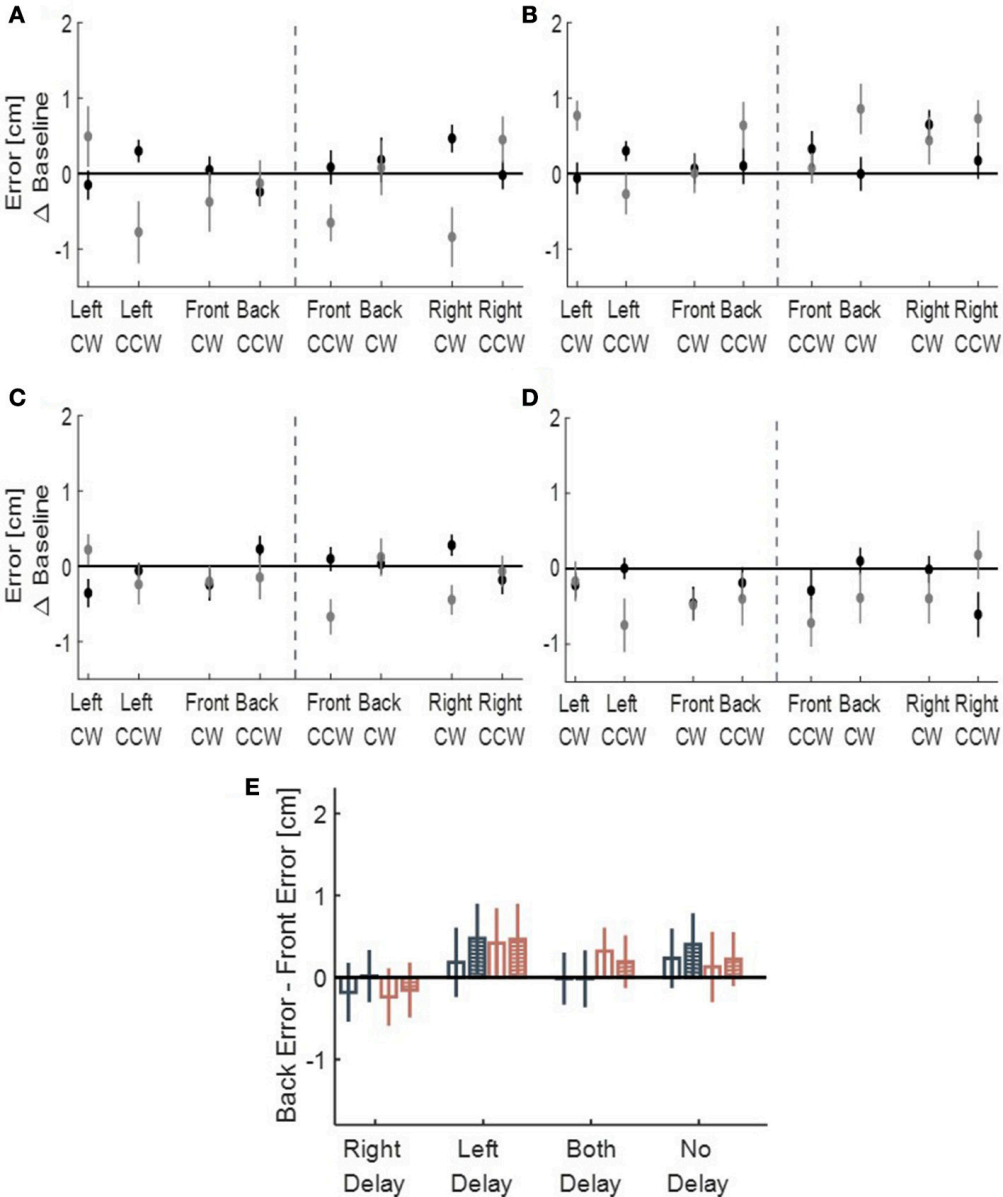
Deviation of the drawn circles toward front (gray) and back (black) directions, divided to left and right initiation (dashed line), for **(A)** Left delay group, **(B)** Right delay group, **(C)** Both delay group and **(D)** No delay group. The X-axis represents the location of the drawn circle (front, back, right, and left) and the direction of clockwise (CW) and counterclockwise (CCW). No pattern of deviation is observed in those directions. **(E)** Statistical analysis of Back error - Front error Difference for the circles initiated in the left (dark blue) and in the right (light red). Empty bars are for Late Adaptation session and bars with stripes are for Washout session. From the graph, no similar pattern of elongation toward front or back is observed.

We performed another control analysis on the drawings of participants from all four conditions (LD, RD, BD, and ND)—we calculated the front and back deviation from ideal circles. There were no consistent elongation to the front and to the back of neither right- or left-initiated circles (Figure 8), suggesting that the transfer effect was specific to the lateral dimension of movement. However, in our experimental setup, movements toward front and back directions were partly constrained. Therefore, to fully assess the effect of asymmetric delay on movements in these directions, further experiments are required.

From these results we conclude that after adapting to a visuomotor delay between the movement of the hand and its visual feedback in either or both left or the right workspaces, participants presented aftereffects in reach movements to the workspace in which the delay was presented, consistent with context-dependent adaptation. They also exhibited transfer to blind drawing that caused spatial elongation of the drawing, and the pattern of elongation along the frontal plane depended on the workspace in which the delay was presented—left and both delay caused asymmetrical elongation only to left initiated circles and right delay caused symmetrical elongation to both left and right initiated circles. This shows that exposure to delay might be processed differently according to the workspace in which it was presented, and that the laterality in the visual feedback is important for shaping our representation of the environment when adapting to temporal misalignment between the different sensory streams. Importantly, between the models that we simulated, this pattern of transfer is only consistent with the perceptual and motor asymmetry model.

## Discussion

In this study, we set out to establish the link between spatial representation of information across workspaces and adaptation to temporal misalignment between the senses. We computationally modeled and experimentally validated the effect of delayed visual feedback of cursor movement that is presented exclusively in one or in both workspaces on participants' movements with and without visual feedback. Consistent with previous studies, the behavioral results show that following an exposure to a visuomotor delay either in one or both workspaces, participants modified the extent of their reaching movements: the abrupt presentation of the delay caused hypermetria—participants made larger reaching movements; they reduced this hypermetria throughout adaptation, and exhibited aftereffects in the workspace where the delay was applied. This means that to reduce the overshoot of the target, participants compensated for the changes in the visual feedback by constructing an internal representation of the perturbation that was specific to the workspace it was applied in. Importantly, the effects of asymmetric delay in the left and right workspaces mirrored each other.

In contrast, transfer of adaptation to the blind circle-drawing task revealed a different picture. Following adaptation to visuomotor delay, we observed hypermetric circles that were elongated only in one side. Whether the circles were hypermetric dependent on the workspace where the drawing was initiated (left or right) and on the workspace in which the delay was presented (left, right or both). The effect of the workspace of drawing initiation on the side of the circle that was hypermetric was demonstrated most clearly in the circles that were drawn in the front and the back locations. Although these circles were all in the middle of the task space, the drawings were different depending on the workspace where they were initiated.

Interestingly, the hypermetria in the drawings was different between the left delay, right delay, and both delay groups. Adaptation to left delay or delay in both workspaces caused elongation of only leftward blind drawings. In contrast, adaptation to right delay caused elongation in both directions. A simulation study confirmed that simple generalization without laterality effect cannot explain these findings. Instead, we had to include an asymmetrical, workspace-dependent, transfer of adaptation. The pattern of asymmetry was not consistent with an asymmetrical transfer model that is based exclusively on perceptual and motor asymmetry, but rather required the combined effect of laterality in perception and action. We concluded that visuomotor delay might be processed differently depending on the workspace in which it was presented, and we further suggest that this difference resulted from *Perceptual-Motor Asymmetry* between the hemispheres.

### Adaptation and representation of visuomotor delay

Visuomotor delay was investigated in various types of movements, such as driving (Cunningham et al., 2001), tracking (Foulkes and Miall, 2000; Leib et al., 2017), and reaching (Botzer and Karniel, 2013). However, the effect of asymmetrical visuomotor delay was not investigated. One exception is a recent study in which participants were exposed to visuomotor delay while performing a complex task of Pong game in one side of the task space. The effect of the delay was examined by reaching movements with no visual feedback performed at the other side. The results of this study showed asymmetrical generalization from left to right but not from right to left (Farshchian et al., 2018). In our study, we found evidence for initial generalization in the reaching movements toward the opposite direction: when the perturbation was first applied, the participants under-reached the target in movements toward the non-delayed side. This initial generalization was consistent between the left and right workspace specific delay groups. However, after adaptation, no aftereffects were observed in movements toward the non-delayed side in both groups. We believe that our results do not contradict the mentioned study findings: in the Farshchian study, participants played and adapted to the delay only in one workspace, and after adaptation, they were examined for aftereffects in the other workspace. In contrast, in our study, the participants adapted and examined for aftereffects in the entire workspace, but with the presence of delay in movements toward only one workspace.

We found that the effect of adaptation to asymmetric delay during a reaching task transferred to the blind circle drawing task. These circle-drawing movements can be considered as rhythmic movement, which are considered significantly distinct from discrete reaching movement in various aspects (Spencer et al., 2003; Buchanan et al., 2006; Hogan and Sternad, 2007). Therefore, our results are consistent with a study that showed transfer of adaptation to visuomotor delay between reaching movements to out-and-back rhythmic movements and vice versa (Botzer and Karniel, 2013). Furthermore, transfer of adaptation to delayed visual feedback during reaching task to rhythmic movements without visual feedback was also observed (Botzer and Karniel, 2013). Our results are also in agreement with previous results that showed transfer of adaptation to visuomotor rotation during discrete reaching movements to rhythmic slice movements (Scheidt and Ghez, 2007).

In the effort to understand how the brain copes with the inherent delay between the senses, it is well accepted that the brain uses forward models that estimate the outcome of the movement from an efferent copy of the motor command. These forward models were suggested to be formed in the cerebellum (Wolpert et al., 1998; Miall et al., 2007) given the evidence for its role in timing of movements (Ivry, 1996; Spencer et al., 2003), the compensation for circuit delays (Suvrathan et al., 2016), and in the scaling of the muscular action (Diener and Dichgans, 1992). In addition, the cerebellum is important for adaptation from sensory prediction errors, i.e., the difference between the predicted and the actual sensory feedback (Taylor et al., 2010; Morehead et al., 2017). It is likely that the cerebellum is involved in adapting movement amplitude when exposed to visuomotor delay, but further investigation is needed to directly examine this hypothesis.

The jury is still out on the question how delay is represented in the motor system. Adaptation to delayed information can be obtained by representing the perturbation as time-based or state-based. On one hand, recent studies provided support for time-based representation of delayed feedback (Witney et al., 1999; Levy et al., 2010; Rohde et al., 2014; Leib et al., 2015; Avraham et al., 2017b). In contrast, other studies provided evidences for state-based representation, and that participants were not able to correctly represent the delay as time difference. For example, adding a delay to force feedback affects stiffness perception (Pressman et al., 2007; Nisky et al., 2008, 2010, 2011; Di Luca et al., 2011). Other example comes from the effect of visuomotor delay on movements during adaptation and its transfer (Botzer and Karniel, 2013; Avraham et al., 2017a). This suggest that humans are not able to perceive the delay as time difference between the sensory inputs, and therefore, are unable to realign the different sensory inputs to avoid perceptual biases. Our results are inconsistent with a time-based representation—the participants modified their movements' extent following exposure to delay, and exhibited aftereffects when the delay was unexpectedly removed—if they would represent the delay as time difference they would have modified the timing of their movements rather than the amplitude.

Once agreed on a state-based representation, which one is used? One possible representation of delay is modification of mass estimation when interacting with robotics arm (Farshchian et al., 2018). This representation cannot be used in our case, as the construction of robotic arm used in our experiment was symmetric. In addition, it was suggested that the misalignment between the hand and the cursor is interpreted as a mechanical load of mass (the cursor) with a spring and a damper that connects between the hand and the cursor. This model was used to explain the changes in grip forces accompanied with delayed visual feedback (Sarlegna et al., 2010), the changes in resistive sensation following adaptation to visuomotor delay (Takamuku and Gomi, 2015), and the generalization between adapting to a visuomotor delay or to a mechanical system between the hand and the cursor (Leib et al., 2017). Another possible state-based representation of visuomotor delay is considering an increase in gain between the hand and the cursor (Avraham et al., 2017a). Both mechanical system and gain representation can be used to explain the hypermetria in our results. Therefore, for simplicity of implementation and interpretation, in our computational model we used the simple gain representation of the delayed visual feedback. Using this gain representation, we were able to simulate the results observed in our experiment both in reaching and blind drawing tasks. However, this particular choice is not critical in our current work, and any remapping that could reproduce elongated reaches and circles could be used to demonstrate the predictions of the different laterality effects.

### On the other hand?

It is potentially interesting to repeat our experiments with the left hand of either right- or left-handed individuals. However, right-handed individuals use additional cognitive structures outside of the motor system to learn a motor task with the left hand (Grafton et al., 2002). Therefore, examining adaptation to delay with the left hand is not likely to provide a substantial contribution to the validation of our model. Furthermore, testing our model with left-handed participants may also be of limited value for testing our current hypotheses as there are many differences between left and right handed, as demonstrated in the evidence that the cerebral organizations of the hemispheres are not mirror images of each other (Wolff et al., 1977). Such differences were observed in the functional connectivity between motor areas in the two hemispheres in a resting state, which was significantly higher for right handed participants (Pool et al., 2015). This functional connectivity between the hemispheres in right handed may play an important role in learning lateralized perturbation such as the one presented in our study. Therefore, we think that it is interesting to study left-handed individuals, but it is outside of the scope of the current study.

### Right hemisphere dominance and a model for laterality in the processing of visuomotor delay

When faced with an imbalanced stimulation across space, the hemispheres demonstrate different patterns of activation and inhibition, and these are reflected in asymmetric attention, perception, and action across workspaces (Reuter-Lorenz et al., 1990). An example of an asymmetric perception in healthy individuals is leftward perceptual bias—a spatial deviation toward stimuli located on the left side. This bias was suggested to arise from asymmetries in hemispheric activation: the left hemisphere is activated only by stimuli in the right hemispatial field, while the right hemisphere is activated in response to stimuli in both the left and the right hemispatial fields (Heilman and Valenstein, 1979). In addition, the right hemisphere can also interact more strongly with the left hemisphere, by exerting inhibition activity over cortical areas in the left hemisphere (Koch et al., 2011; Gotts et al., 2013). Because the activation process in the right hemisphere occurs in different locations for right or left stimuli (Corbetta et al., 1993), it is possible that the inhibition activity from right to left will only take place in response to left stimuli. Regarding to the control of right hand movements in right handers, it is well known that the left hemisphere controls movements toward both workspaces. However, studies suggested that the right hemisphere is involved in right-hand movements only toward the left workspace (Farnè et al., 2003; Heilman and Valenstein, 2010). This explains why in the case of processing delayed visual feedback in our experiment, leftward movements with the right hand can be strongly affected also by the right hemisphere.

Although we were unable to fully control the participants' gaze direction, and to maintain their middle visual field fixed at the mid-point location, we received strong evidence that our results cannot be attributed solely to the effect of delay on the sensorimotor system without considering the differences between the hemispheres. Based on both of our computational model and experimental results, we suggest that exposure to delay excites motor circuits associated with movement extension in the relevant hemisphere, such that: (1) Delay only in the left workspace has an excitatory effect on brain areas responsible for movement extension in the right hemisphere (Figure 1B). Therefore, an exposure to delay only in the left visual field causes only leftward hypermetria (Figure 1A). (2) Delay in the right workspace affects both hemispheres (Figure 1B), resulting in transfer of hypermetria toward both workspaces (Figure 1A). (3) Delay in both workspaces excites motor areas in both hemispheres. However, as a result of exposure to left delay, the right hemisphere inhibits the left, and cancels the excitatory effect of delay (Figure 1B). Overall, excitation effect is only maintained in the right hemisphere, thereby affecting leftward movements performed without visual feedback and causing leftward hypermetria.

In the current study, we coupled between movement direction and the hemispace toward which the movement is performed. This is because we wanted to understand the basis of the adaptation to asymmetrical delay, without having to consider multiple factors. Future studies should investigate the effect of decoupling these two factors.

The asymmetrical leftward hypermetria in the drawings of the participants can be related to the recently reported asymmetrical expansion of drawings in patients with right brain damage, which is known as “hyperschematia.” This disorder affects the representation of extra-personal space, resulting in left asymmetric expansion both when copying an object or drawing from memory (Rode et al., 2014). In our study, participants' drawings without visual feedback were asymmetrically leftward elongated after adaptation to left delay and delay in both sides.

The observed pattern of activation and inhibition in the hemispheres can also potentially explain some motor impairments that involve asymmetrical perception and action, such as the motor aspects of Hemispatial Neglect. Neglect patient may exhibit unilateral temporal disorders of slowness in initiation and execution of movements (directional hypokinesia and directional bradykinesia, respectively), and unilateral spatial disorders of reduction in movement amplitude (directional hypometria) (Mattingley et al., 1994). In light of motor impairments such as neglect, previous studies proposed a model to explain the imbalance between the hemispheres (Heilman and Valenstein, 2003). In this study, the authors argued that the asymmetry in perception and intention between the hemispheres is a result of asymmetrical representation of the workspaces, such that the right hemisphere incorporates representations for both workspaces, yet the left hemisphere holds representation only for the right workspace. However, in addition to the spatial deficit observed in neglect, several studies also reported time-related impairments. For example, reports of a considerable delay in visual awareness of left stimuli compared to right stimuli (Robertson et al., 1998). Previous studies suggested that neglect is a spatial-temporal rather than a purely spatial deficit (Becchio and Bertone, 2006), and that there is a link between laterality and temporal aspects of information processing. We show here that after an exposure to asymmetrical delay, healthy participants exhibit hypermetric asymmetrical movements. Although the participants exhibited hypermetria rather than hypometria, we believe that this spatial asymmetry can be related to the mechanisms underlying the spatial disorders in neglect. Hence, we suggest that the imbalance between the hemispheres can also be associated with visuo-temporal processes. However, further research is needed in order to ascertain this possibility.

The observed connection between time and space, demonstrated through our model, can help to explain the motor deficits observed in neglect, which has been suggested to be associated with distortions in time processing (Becchio and Bertone, 2006). By integrating the model for unilateral neglect with our proposed model, we can further establish the connection between temporal perturbations and spatial-motor impairments. Understanding the role of each hemisphere in mediating time and space representation can provide important insights on pathological cases involving injury in only one side of the brain and also to provide new directions for diagnosis and rehabilitation.

## Author contributions

CA, GA, and IN designed the experimental protocol and hypotheses. CA performed the experiments. CA, GA, FM-I, and IN analyzed the data, interpreted the results and wrote the paper.

### Conflict of interest statement

The authors declare that the research was conducted in the absence of any commercial or financial relationships that could be construed as a potential conflict of interest.
